# Pathophysiological mechanisms and clinical management of type 2 diabetes mellitus complicated with anal fistula

**DOI:** 10.3389/fsurg.2026.1776450

**Published:** 2026-04-10

**Authors:** Linhui Li, Shuangxi Zhang

**Affiliations:** 1 Department of Anorectal Diseases, The First Affiliated Hospital of Henan University of Traditional Chinese Medicine, Zhengzhou, China; 2 The First Clinical Medical College of Henan University of Traditional Chinese Medicine, Zhengzhou, China

**Keywords:** anal fistula, blood glucose control, pathophysiological mechanism, regenerative medicine, sphincter-preserving surgery, type 2 diabetes mellitus (T2DM)

## Abstract

**Background:**

The comorbidity of type 2 diabetes mellitus (T2DM) and anal fistula is a prevalent global clinical challenge. Anal fistula is the second most common anorectal disease, with an incidence 1.81–2.01 times higher in diabetic patients than in the general population. Diabetic patients face elevated risks of postoperative infection, delayed healing, and recurrence, closely linked to poor preoperative glycemic control. Current strategies are mostly extrapolated from general population guidelines, failing to address the unique metabolic, immune, and microcirculatory abnormalities in this group, leading to suboptimal outcomes.

**Methods:**

This narrative review followed PRISMA 2020 guidelines. We systematically searched PubMed, Embase, Cochrane Library, and CNKI for literature (2018–June 2024) on T2DM complicated with cryptoglandular anal fistula, including clinical studies, high-quality animal experiments, and systematic reviews. Exclusion criteria: type 1/gestational diabetes, Crohn's-related fistulas, case reports (*n* < 10). We synthesized evidence on surgical optimization, regenerative medicine, and perioperative glycemic management.

**Results:**

① Sphincter-preserving surgeries (LIFT, VAAFT, TROPIS, EAF) reduce incontinence risk; TROPIS achieved 87.6% long-term healing in diabetics, while EAF is well-established for complex fistulas. ② Regenerative therapies (CGF, MSCs) promote healing; autologous MSC-based therapies yielded 68.4%–84.6% healing for complex fistulas, with superior safety/operability vs. allogeneic products. ③ Preoperative HbA1c < 7.0% reduced infection to 8.2%, with perioperative glucose targets of 140–180 mg/dL optimal; once-weekly insulin icodec improved compliance.

**Conclusions:**

Individualized multidisciplinary strategies tailored to fistula complexity and glycemic status are essential. Future large-scale RCTs in diabetic patients are needed to validate novel biomaterials and anti-inflammatory agents to optimize outcomes.

## 1 Introduction

Diabetes is a globally prevalent metabolic disease, with a substantial disease burden on cardiovascular system and surgical prognosis (1). Anal fistula is the second most common anorectal disease, accounting for 1.67%–3.60% of anorectal diseases in China (2) and 8.00%–25.00% in Western countries (3). The comorbidity of T2DM and anal fistula presents dual clinical challenges of “elevated incidence + refractory healing”: the incidence of anal fistula in diabetic patients is 1.81–2.01 times that in the general population (4). Globally, approximately 537 million adults aged 20–79 years are living with diabetes, with prevalence projected to reach 783 million by 2045, which will further increase the clinical burden of diabetes-related anal fistula (5).

Diabetic patients have a significantly elevated risk of poor postoperative outcomes after anal fistula surgery: ① Delayed wound healing: the risk of delayed wound healing (defined as incomplete epithelialization or persistent exudation 6–12 weeks after surgery) is 2.5 times higher in diabetic patients than in non-diabetic patients (OR = 2.5, 95% CI 1.8–3.4) (6). ② High recurrence risk: diabetes significantly increases the risk of postoperative anal fistula recurrence (35.7% vs. 10.4% in non-diabetic patients, OR = 4.74, 95% CI: 1.42–15.79, *P* = 0.018) (7). ③ Low healing rate: only 50% of T2DM patients with anal fistula achieved complete wound healing within 35 days after surgery in a single-center retrospective cohort study (*n* = 244) (8).

However, current clinical diagnosis and treatment protocols for this population are mostly extrapolated from guidelines for the general population, without fully accounting for the unique metabolic, immune, and microcirculatory characteristics of diabetic patients. Therefore, clarifying the pathophysiological link between T2DM and anal fistula, and integrating evidence-based, individualized treatment strategies are critical to improving patient prognosis.

## 2 Review scope and methodology

This narrative review focuses on the core theme of “type 2 diabetes mellitus complicated with cryptoglandular anal fistula”, aiming to synthesize existing diagnostic and therapeutic evidence and provide references for clinical decision-making. The review was conducted and reported in compliance with the PRISMA 2020 statement for systematic reviews (9).

### 2.1 Literature search strategy

We systematically searched four databases: PubMed, Embase, Cochrane Library, and CNKI, for literature published between January 2018 and June 2024. The search terms included combinations of: “diabetes mellitus”, “type 2 diabetes mellitus (T2DM)”, “anal fistula”, “perianal fistula”, “surgical treatment”, “regenerative medicine”, and “blood glucose control”. The initial search yielded a total of 1,286 records (PubMed: 327, Embase: 412, Cochrane Library: 89, CNKI: 458). For Section 4.4 (Economic Analysis), the search criteria were expanded to include seminal cost-effectiveness studies published outside the primary 2018–2024 timeframe to ensure comprehensive economic evidence (10, 11).

### 2.2 Inclusion and exclusion criteria

**Inclusion criteria**: ① Study types: human clinical studies (retrospective cohort studies, prospective studies, RCTs), high-quality animal experiments (sample size ≥10 per group with clear control design), and systematic/narrative reviews in Chinese or English; ② Study subjects: patients with a confirmed diagnosis of T2DM complicated with cryptoglandular anal fistula (or corresponding T2DM + anal fistula animal models); ③ Core outcomes: healing rate, recurrence rate, anal function (incidence of incontinence), and infection risk.

**Exclusion criteria**: ① Study subjects: patients with type 1 diabetes, gestational diabetes, Crohn's disease-related anal fistula, or tuberculous anal fistula; ② Study types: case reports (sample size <10), duplicate publications, and conference abstracts without complete data; ③ Outcome indicators: studies not reporting healing, recurrence, or blood glucose-related outcomes.

### 2.3 Literature screening process

**Figure 1:**
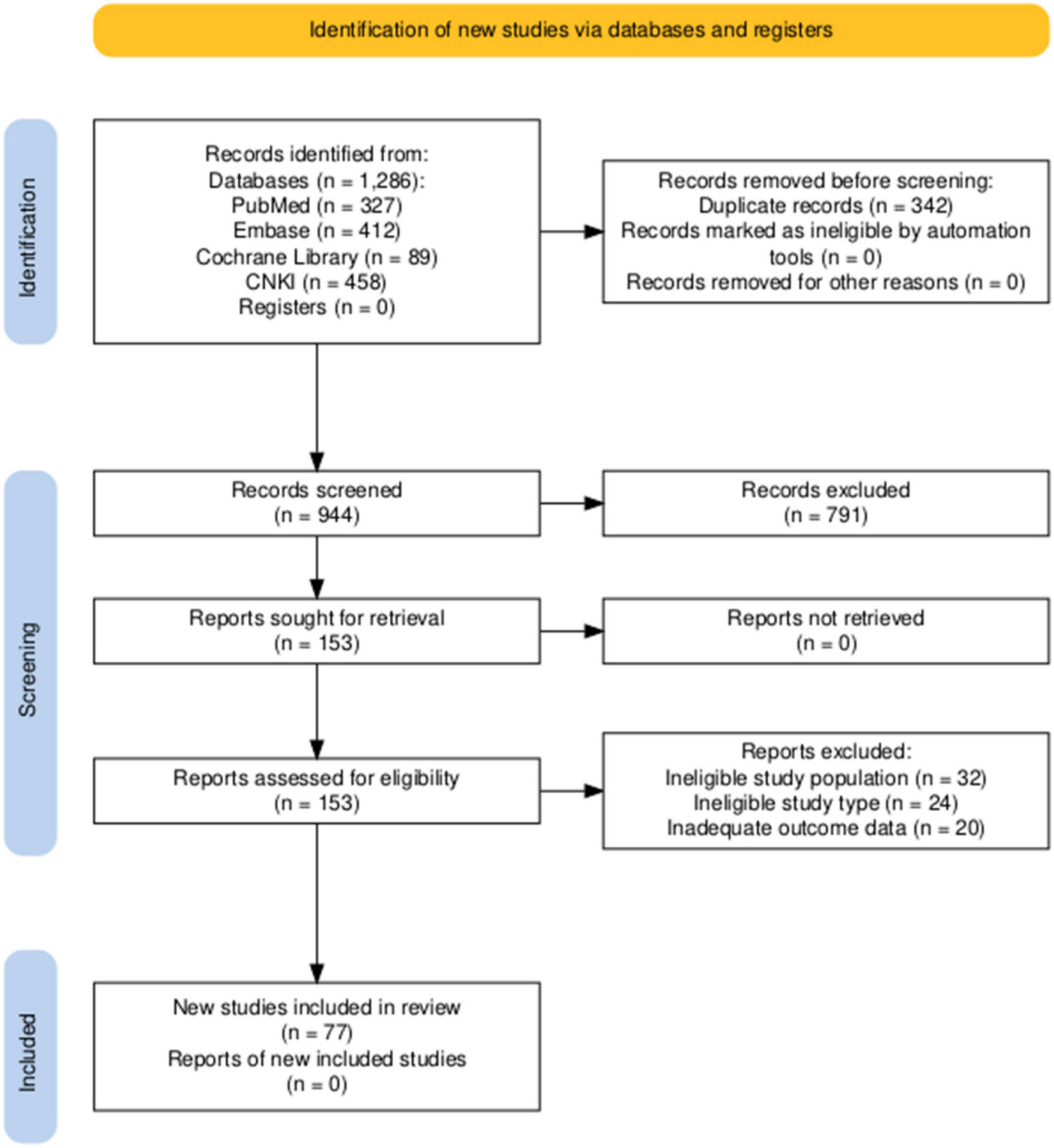
PRISMA 2020 flow diagram of literature screening process.

After removing 342 duplicate records, 944 records remained for title and abstract screening. Of these, 791 records were excluded for not meeting the inclusion criteria, leaving 153 records for full-text assessment. After full-text review, 76 records were excluded for failing to meet the eligibility criteria, resulting in a final total of 77 studies included in this review. The detailed screening process is presented in Figure 1, which was developed in accordance with the PRISMA 2020 flow diagram standard (9).

This flow diagram illustrates the systematic literature screening process of this narrative review, which was conducted in full compliance with the PRISMA 2020 statement (9). A total of 1,286 records were initially identified from 4 databases (PubMed, Embase, Cochrane Library, CNKI) for literature published between January 2018 and June 2024. After duplicate removal, title/abstract screening, and full-text eligibility assessment, 77 studies were finally included in this review. Two additional cost-effectiveness analyses (10, 11) were specifically retrieved to support the economic evaluation in Section 4.4, bringing the total evidence base to 79 studies. Abbreviations: PRISMA, Preferred Reporting Items for Systematic Reviews and Meta-Analyses; T2DM, type 2 diabetes mellitus; RCTs, randomized controlled trials.

## 3 Pathophysiological mechanisms linking T2DM and anal fistula

**Figure 2:**
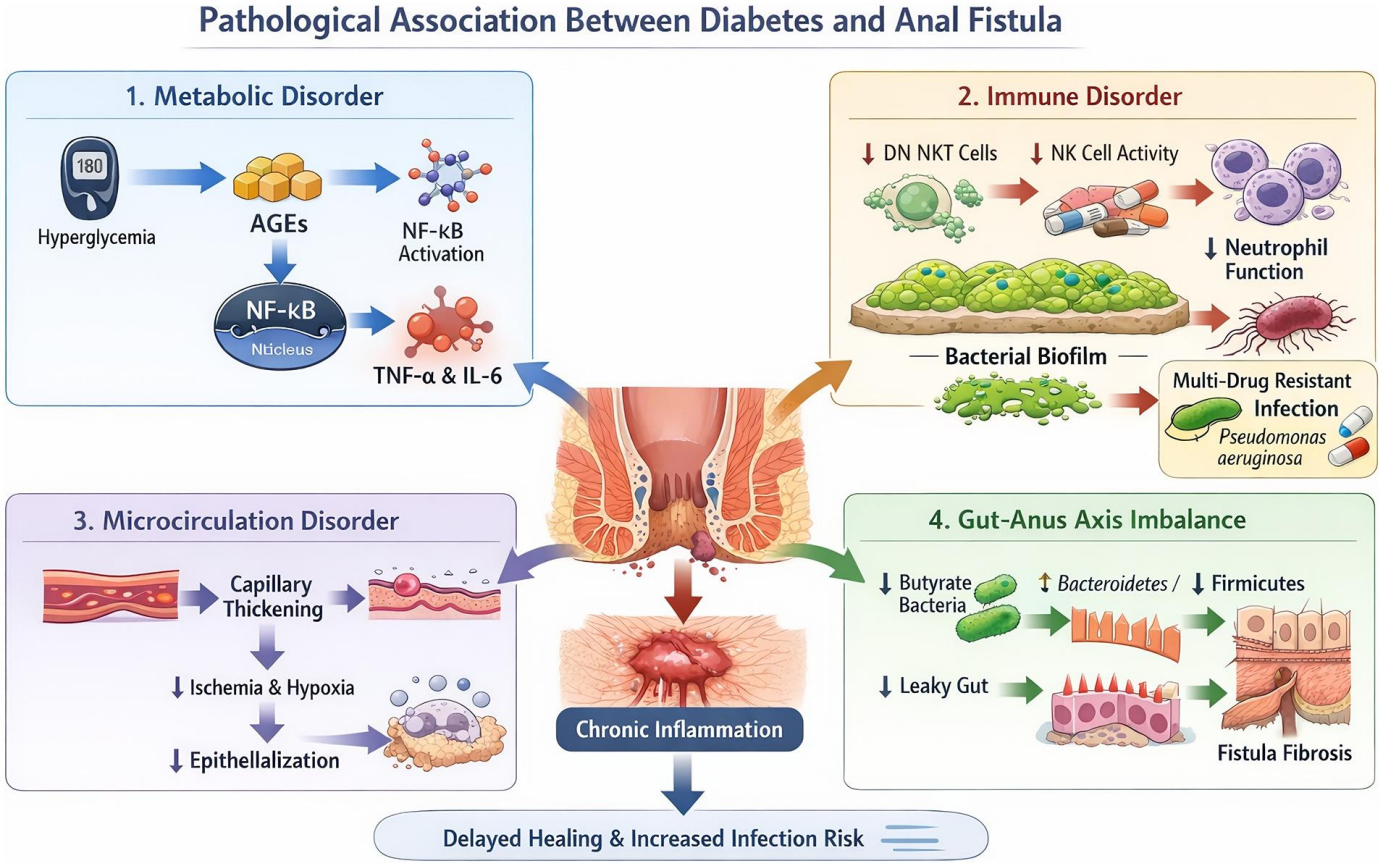
Pathological Association Between Diabetes and Anal Fistula. Schematic representation of four core pathological mechanisms linking type 2 diabetes mellitus (T2DM) to the development and progression of anal fistula: Metabolic Disorder: Hyperglycemia promotes advanced glycation end products (AGEs) formation, which activates nuclear factor-κB (NF-κB) signaling, leading to the production of pro-inflammatory cytokines (TNF-α, IL-6) and chronic inflammation. Immune Disorder: Diabetes impairs innate immune function (reduced DN NKT cells, NK cell activity, and neutrophil function), facilitating bacterial biofilm formation and multi-drug resistant Pseudomonas aeruginosa infection, thereby exacerbating local inflammation. Microcirculation Disorder: Chronic hyperglycemia induces capillary thickening, resulting in tissue ischemia and hypoxia, which impairs epithelialization and delays wound healing. Gut-Anus Axis Imbalance: T2DM-associated gut dysbiosis (decreased butyrate-producing bacteria, reduced Firmicutes/Bacteroidetes ratio) and intestinal barrier dysfunction (“leaky gut”) promote systemic inflammation and fistula fibrosis. Collectively, these mechanisms converge to create a pro-inflammatory microenvironment, increasing infection risk and delaying the healing of anal fistula in diabetic patients.

The impaired wound healing and elevated fistula risk in diabetic patients are driven by four interrelated pathophysiological pathways, as detailed below and illustrated in Figure 2.

This figure summarizes the four interrelated core pathways driving elevated fistula onset risk and impaired postoperative wound healing in T2DM patients: ① Metabolic disorder: hyperglycemia-induced advanced glycation end products accumulation activates NF-κB signaling, triggering excessive pro-inflammatory factor release and persistent inflammation; ② Immune dysfunction: impaired innate immune function promotes bacterial biofilm formation and multidrug-resistant infection; ③ Microcirculation impairment: capillary basement membrane thickening causes tissue ischemia and hypoxia, inhibiting epithelialization; ④ Gut-anus axis imbalance: gut microbiota dysbiosis damages the intestinal mucosal barrier, accelerating fistula fibrosis and chronic inflammation. These pathways jointly lead to delayed healing and elevated infection/recurrence risk in T2DM patients with anal fistula. Abbreviations: T2DM, type 2 diabetes mellitus; AGEs, advanced glycation end products; NF-κB, nuclear factor-kappa B; TNF-α, tumor necrosis factor-alpha; IL-6, interleukin-6.

### 3.1 Metabolic disorder: the initiator of persistent inflammation

Chronic hyperglycemia leads to the accumulation of advanced glycation end products (AGEs), which activates the NF-κB signaling pathway in the nucleus (12). This results in excessive release of pro-inflammatory factors including TNF-α and IL-6, which inhibits granulation tissue formation and prolongs the inflammatory response, clinically manifesting as persistent exudation from the anal fistula wound and delayed healing (13).

### 3.2 Immune dysfunction: increased susceptibility to infection

Diabetic patients exhibit specific depletion of double-negative (DN) NKT-like cells (CD4^−^CD8^−^) and decreased NK cell activity, which directly impairs the innate immune response to perianal infection (14). This further impairs the chemotactic and phagocytic functions of neutrophils, promotes bacterial biofilm formation on the fistula tract wall (15), increases the risk of infection with multidrug-resistant bacteria (e.g., *Pseudomonas aeruginosa*), and limits the efficacy of antibiotic therapy (16).

### 3.3 Microcirculation impairment: compromised tissue repair

Hyperglycemia induces thickening of the capillary basement membrane, leading to ischemia and hypoxia in perianal tissues (17). This inhibits keratinocyte proliferation and epithelialization of the surgical wound, clinically manifesting as a 30%–50% prolongation of postoperative healing time in diabetic patients (18).

### 3.4 Gut microbiota dysbiosis: the “gut-anus axis” link

Diabetic patients exhibit a reduction in butyrate-producing bacteria and an abnormal Bacteroidetes/Firmicutes ratio in the gut (19, 20), which damages the intestinal mucosal barrier, promotes bacterial translocation to the perianal region, and accelerates fistula tract fibrosis and persistent inflammation (21). Probiotic intervention has been shown to reduce fistula recurrence risk in clinical studies, providing indirect validation of this mechanism (22).

## 4 Evidence-based treatment strategies for T2DM complicated with anal fistula

The management of diabetes complicated with anal fistula faces dual core challenges: on the one hand, hyperglycemia-induced microvascular lesions, immune dysfunction, and inflammatory disorders delay wound healing (23–25); on the other hand, the anatomical complexity of anal fistula (especially complex high fistulas) increases the risk of surgical failure and anal incontinence (26–28). Current treatment strategies focus on sphincter-preserving surgical optimization, regenerative medicine adjuvant therapy, and standardized perioperative glycemic management, with an emphasis on multidisciplinary collaborative care.

### 4.1 Advances in surgical treatment

#### 4.1.1 Limitations of traditional surgical procedures

As the classic treatment modalities for anal fistula, fistulectomy and fistulotomy with cutting seton aim to completely eradicate the infected focus or achieve cure through chronic cutting and drainage. However, their inherent limitations are significantly amplified in diabetic patients, as detailed below.

(1) *Fistulectomy*

Fistulectomy involves complete *en bloc* excision of the fistula tract, creating an open wound that heals via secondary intention (granulation tissue growth from the wound base upward).

The main limitations in diabetic patients are as follows:

① Large wound defect and prolonged healing time: Complete fistula excision results in a large open wound. Even in non-diabetic patients, this procedure is associated with a long healing period, which is further prolonged and unpredictable in diabetic patients with impaired wound healing capacity. A prolonged healing course requires patients to endure persistent pain, activity limitations, and frequent dressing changes.
② Significantly elevated risk of postoperative infection: The large open wound provides an ideal environment for bacterial colonization and invasion. Under the combined effects of impaired immune function and hyperglycemia-induced bacterial growth promotion in diabetic patients, the risk of postoperative wound infection is extremely high (29).
③ Non-negligible risk of anal incontinence: For fistulas involving a large portion of the internal and external anal sphincters, complete fistula excision inevitably causes significant sphincter damage, leading to varying degrees of anal incontinence (including gas leakage, soiling, and even fecal incontinence) (30). For diabetic patients with pre-existing diabetic peripheral neuropathy, even minor additional sphincter damage can have catastrophic consequences for quality of life.

(2) *Fistulotomy with Cutting Seton*

Fistulotomy with cutting seton involves passing a non-absorbable suture (seton) through the fistula tract, with gradual tightening over time. Through mechanical compression and foreign body-induced fibrosis, the seton slowly cuts through the overlying sphincter muscle while inducing fibrosis and healing of the posterior tissue.

The main limitations in diabetic patients are as follows:

① Extremely long treatment cycle and persistent infection risk: The “slow cutting” process of the cutting seton typically takes several weeks to months to complete. During this period, the fistula tract remains open for drainage, and the seton itself acts as a foreign body that can harbor bacterial colonization. For diabetic patients, this long-term open wound and chronic inflammatory state greatly increase the risk of secondary infection and abscess formation.
② Significant pain and discomfort: The presence of the seton and each tightening procedure cause significant pain and foreign body sensation, severely impairing daily activities and quality of life. This long-term negative experience adds additional physical and psychological burden to diabetic patients, who require strict glycemic control and stable lifestyle habits.
③ Persistent risk of anal incontinence: Although the theoretical basis of the cutting seton is “cutting while healing” to reduce the risk of complete sphincter rupture, it still causes iatrogenic cutting injury to the sphincter. Multiple studies have confirmed that cutting seton is associated with an increased risk of sphincter damage and anal incontinence (31, 32). For diabetic patients with pre-existing fragile sphincter function, this progressive but definite injury has unpredictable long-term impacts on anal continence.
④ Unfavorable cost-effectiveness: The long treatment cycle requires multiple outpatient visits for seton tightening and wound care, which increases patients' time, transportation, and cumulative medical costs.

#### 4.1.2 Optimized and innovative surgical techniques: sphincter-preserving procedures as the core strategy

In response to the impaired tissue repair capacity and the critical need for anal function preservation in diabetic patients, sphincter-preserving surgical techniques and minimally invasive procedures have become the research focus and clinical priority. The core features, advantages, limitations, healing rates, and applicable populations of common sphincter-preserving procedures are detailed in Supplementary Table 1 (78–82). The key evidence for each technique is summarized below:

##### 4.1.2.1 Core sphincter-preserving techniques with established efficacy

① ***Ligation of the Intersphincteric Fistula Tract (LIFT)***: LIFT involves ligation and division of the fistula tract within the intersphincteric plane, with complete preservation of the external anal sphincter. It has a reported overall healing rate of 70%–85% in complex anal fistulas, with an incontinence risk <5% (33, 34). However, in diabetic patients, hyperglycemia-induced microcirculatory disturbance and impaired tissue repair may increase the recurrence rate by 10%–15% compared to non-diabetic populations (Supplementary Table 1). For diabetic patients, its minimal tissue trauma and sufficient drainage make it a preferred first-line option for simple transsphincteric fistulas.
② ***Endorectal Advancement Flap (EAF)***: EAF is a well-established sphincter-preserving technique that involves raising a full-thickness flap of rectal mucosa, submucosa, and circular muscle to cover and close the internal opening of the fistula, with complete preservation of the anal sphincter complex. It has a short-term healing rate of 60%–80%, with a long-term recurrence rate of 20%–40% (31, 35) in complex anal fistulas, with an incontinence risk <10% in experienced centers [31, 35). For diabetic patients, EAF avoids open perianal wounds, reduces postoperative pain and infection risk, and is particularly suitable for patients with recurrent fistulas or high risk of incontinence. Note: Chronic hyperglycemia significantly increases the risk of flap ischemia, necrosis, and dehiscence in diabetic patients, potentially reducing the actual healing rate by 15%–20% compared to reported values (Supplementary Table 1).
③ ***Video-Assisted Anal Fistula Treatment (VAAFT)***: VAAFT is a minimally invasive procedure that uses a fistuloscope to visualize the entire fistula tract, allowing precise debridement of the infected tract and closure of the internal opening under direct vision. It has an overall healing rate of 75%–85%, with a postoperative anal incontinence incidence <5%, showing significant advantages in anal function preservation (35–37). It is suitable for complex high fistulas with multiple branches in diabetic patients, as it minimizes unnecessary tissue damage and avoids residual infected foci (82).
④ ***Transanal Opening of Intersphincteric Space (TROPIS)***: TROPIS involves opening the intersphincteric space via a transanal approach to debride the infected cryptoglandular focus, with partial division of the internal anal sphincter. It has achieved a long-term healing rate of 87.6% in diabetic patients (the only procedure with clear diabetic-specific efficacy data), showing favorable curative effects (38–40). However, it should be noted that although TROPIS is classified as a sphincter-preserving procedure, it requires partial division of the internal anal sphincter, which may sacrifice a large portion of the internal sphincter in patients with extensive fistula involvement. This increases the risk of postoperative anal continence impairment, especially in diabetic patients with pre-existing neuropathy or baseline sphincter dysfunction. Therefore, strict preoperative assessment of anal function is mandatory before selecting this procedure. The recurrence rate in diabetic patients is 15%–20% higher than in the general population, requiring more meticulous postoperative wound management and glycemic control. Partial internal sphincter division carries a higher risk of permanent continence impairment in patients with diabetic peripheral neuropathy (Supplementary Table 1).
⑤ ***Fistula Laser Closure (FiLaC)***: FiLaC uses a radial laser fiber to ablate the fistula tract epithelium, inducing fibrosis and closure of the tract. It is a minimally invasive, sphincter-preserving procedure suitable for patients at high risk of incontinence. However, due to the higher risk of non-healing and recurrence in diabetic patients, stricter preoperative glycemic control (HbA1c < 7.0%) (40–42) is required for this procedure (41–43).

### 4.2 Adjuvant therapeutic strategies: regenerative medicine and targeted anti-inflammatory intervention

Based on the core characteristics of “persistent inflammation and impaired repair” in diabetic wound healing, regenerative medicine, functional biomaterials, and targeted anti-inflammatory treatments have emerged as important adjuvant therapies for diabetic patients with anal fistula.

#### 4.2.1 Application of regenerative medicine and functional biomaterials

(1) ***Concentrated Growth Factor (CGF)***: CGF is a bioactive material derived from autologous peripheral blood, which releases vascular endothelial growth factor (VEGF) and platelet-derived growth factor (PDGF) to promote angiogenesis and granulation tissue formation. Its anti-inflammatory and pro-healing effects have been confirmed in a porcine anal fistula model (44). Currently, small-sample clinical studies have shown that CGF can significantly shorten wound healing time in diabetic patients after anal fistula surgery, but large-sample RCTs are still needed to verify its long-term efficacy.
(2) ***Functional Hydrogel Biomaterials***: Injectable hydrogels with antibacterial and pro-healing functions have shown favorable application prospects in anal fistula treatment. Tea tree oil-loaded carboxymethyl chitosan hydrogels have confirmed antibacterial effects against common perianal pathogenic bacteria (45), while nanofiber-hydrogel composites can promote granulation tissue ingrowth and fistula closure in animal models (46, 47). For diabetic patients, hydrogel materials can provide a moist healing environment, reduce bacterial colonization, and sustainably release pro-healing factors, which is conducive to promoting wound healing.
(3) ***Natural Product-Derived Adjuvant Therapy***: Multiple natural products with anti-inflammatory and antioxidant effects, including flavonoids and saponins, have been shown to promote diabetic wound healing via regulating the NF-κB and NRF2 signaling pathways (48). These products can be used as oral or topical adjuvant therapies for diabetic patients after anal fistula surgery, but high-quality clinical evidence in the anal fistula population is still lacking.

#### 4.2.2 Cell therapy and targeted anti-inflammatory treatment

(1) ***Mesenchymal Stem Cell (MSC) Transplantation***: MSC transplantation has been approved for the treatment of Crohn's disease-related anal fistula in multiple countries, and has shown favorable efficacy in cryptoglandular anal fistula. It is critical to distinguish between autologous and allogeneic MSC transplantation for diabetic patients:
  ① Autologous MSC-based therapies (most commonly adipose-derived MSCs, ADSCs) have no risk of immune rejection, simple preparation process, and low cost. Clinical studies have shown that autologous ADSC transplantation achieves a short- to long-term healing rate of 68.4%–84.6% in complex cryptoglandular anal fistulas (27, 49, 50), which is particularly suitable for diabetic patients with impaired immune function.
  ② Allogeneic MSC products are commercialized off-the-shelf products, but have extremely high cost, potential risk of immune rejection, and ethical considerations. Existing clinical studies have not verified their safety and efficacy in diabetic patients with anal fistula.
(2) ***Targeted Anti-Inflammatory Intervention***: Persistent inflammation is the core mechanism of fistula non-healing in diabetic patients. Local injection of anti-inflammatory microgels can significantly reduce the levels of TNF-α and IL-6 in the fistula tract, promoting fistula closure in animal models (51). Systemic targeted anti-inflammatory therapies, including Janus kinase (JAK) inhibitors, have shown significant efficacy in closing perianal fistulas in Crohn's disease (52, 53), but their safety and efficacy in diabetic patients with cryptoglandular anal fistula still need to be verified by clinical studies.

### 4.3 Standardized perioperative glycemic management

Glycemic control is the core prerequisite for successful surgical treatment and wound healing in diabetic patients with anal fistula. Uncontrolled hyperglycemia will significantly increase the risk of postoperative infection, delayed healing, and fistula recurrence, so standardized multidisciplinary glycemic management throughout the perioperative period is mandatory.

#### 4.3.1 Glycemic targets for the perioperative period

(1) ***Preoperative Period***: For elective anal fistula surgery, the recommended preoperative glycemic target is HbA1c < 7.0%, and fasting blood glucose <7.0 mmol/L. For patients with HbA1c > 8.5%, elective surgery should be postponed to optimize glycemic control, to reduce the risk of postoperative adverse events (54).
(2) ***Intraoperative Period***: For patients with T2DM undergoing general anesthesia, the recommended intraoperative blood glucose target is 140–180 mg/dL (7.8–10.0 mmol/L). Blood glucose should be monitored every 1–2 h during surgery, to avoid severe hyperglycemia (>10.0 mmol/L) or hypoglycemia (<3.9 mmol/L) (55–57).
(3) ***Postoperative Period***: The recommended postoperative blood glucose target is fasting blood glucose <7.8 mmol/L, and 2-hour postprandial blood glucose <10.0 mmol/L. For critically ill patients in the intensive care unit, the blood glucose target is 140–180 mg/dL. Blood glucose should be monitored at least 4 times a day for patients using subcutaneous insulin injection, to ensure stable glycemic control (54, 58).

#### 4.3.2 Glycemic management regimens for the perioperative period

1. ***Preoperative Glycemic Optimization***: For patients using oral hypoglycemic agents, metformin can be continued until the day of surgery for patients with normal renal function; sulfonylureas, dipeptidyl peptidase-4 (DPP-4) inhibitors, and sodium-glucose cotransporter 2 (SGLT-2) inhibitors should be discontinued 24–48 h before surgery. For patients with poor glycemic control with oral agents, basal-bolus insulin therapy should be initiated 1–2 weeks before surgery to optimize glycemic control (54, 58).
2. ***Long-Acting Insulin Therapy for Improved Compliance***: Once-weekly basal insulin icodec has been approved for the treatment of T2DM in multiple countries, with similar hypoglycemic efficacy to once-daily insulin glargine, and significantly improved treatment compliance (59, 60). For patients undergoing elective anal fistula surgery, once-weekly insulin icodec can reduce the frequency of injection, improve patient compliance, and maintain stable glycemic control throughout the perioperative period.
3. ***Multidisciplinary Collaborative Management***: For patients with complex fistulas and poor glycemic control, a multidisciplinary team (anorectal surgery, endocrinology, clinical pharmacy, nutrition) should be established to develop an individualized glycemic management plan. Nutritional support should be provided throughout the perioperative period, with adequate protein intake to promote wound healing, while avoiding excessive carbohydrate intake that causes blood glucose fluctuations (58). For obese T2DM patients with poor glycemic control despite maximum medical therapy, Roux-en-Y gastric bypass can achieve significant diabetes remission and reduce long-term fistula recurrence risk, which should be comprehensively evaluated 6 months after the initial anal fistula surgery (61).

#### 4.3.3 Adjuvant therapy with hypoglycemic agents with anti-inflammatory effects

Multiple hypoglycemic agents have been shown to have anti-inflammatory and pro-healing effects independent of their hypoglycemic efficacy, which are particularly suitable for diabetic patients with anal fistula:

1. ***DPP-4 Inhibitors (e.g., Sitagliptin)***: In addition to their hypoglycemic effect, DPP-4 inhibitors can inhibit the NF-κB signaling pathway, reduce the levels of pro-inflammatory factors TNF-α and IL-6, and improve endothelial function and microcirculation (62–68). Multiple studies have confirmed that DPP-4 inhibitors can regulate macrophage polarization and inhibit inflammatory pathway activation, which is consistent with the core mechanism of fistula non-healing in diabetic patients (69, 70). Meanwhile, sitagliptin can activate the Nrf2 antioxidant pathway, reduce oxidative stress damage in diabetic wounds, and promote epithelialization (71–75). Multiple preclinical studies have confirmed that sitagliptin has multi-organ protective effects via its anti-inflammatory and antioxidant effects, further supporting its clinical application value in diabetic patients undergoing surgery (76, 77), and it can be used as a preferred hypoglycemic agent for diabetic patients with anal fistula.
2. ***Glucagon-Like Peptide-1 (GLP-1) Receptor Agonists***: GLP-1 receptor agonists have significant anti-inflammatory, antioxidant, and weight loss effects, which can improve insulin resistance and reduce the levels of systemic inflammatory factors (54, 58). For obese diabetic patients with anal fistula, GLP-1 receptor agonists can achieve weight loss and glycemic control simultaneously, which is conducive to reducing postoperative recurrence risk.

### 4.4 Economic analysis and health economic considerations

The primary systematic search identified 77 studies published between January 2018 and June 2024. For the specific economic analysis in this section, two additional seminal foundational studies were included beyond the pre-specified publication time window, given their irreplaceable role in establishing the methodological framework for anal fistula cost-effectiveness evaluation: Adamina et al. (10) provided core cost-effectiveness data comparing anal fistula plug vs. advancement flap for complex anal fistula; and McGlone et al. (11) provided critical evidence on the long-term cost-effectiveness of metabolic surgery in T2DM patients requiring insulin. The FIAT trial (33) previously discussed in Section 4.1.2 also provided high-quality cost-effectiveness data comparing anal fistula plug vs. surgeon's preferred standard treatment.

Exchange rates were retrieved from official central bank databases (European Central Bank and Federal Reserve Bank of St. Louis) in March 2025 (1 USD ≈ 7.18 CNY, 1 EUR ≈ 8.27 CNY), the latest available official data at the time of manuscript submission. All costs are presented in Chinese Yuan (CNY), US Dollars (USD), and Euros (EUR) for international reference.

#### 4.4.1 Cost analysis of surgical procedures

(1) *Traditional Surgical Procedures (Fistulectomy and Cutting Seton)*

Traditional procedures remain the reference standard for simple low-lying fistulas, but carry significant hidden long-term costs in T2DM patients, which are further amplified by hyperglycemia-induced delayed wound healing and elevated complication risks. Adamina et al. (10) demonstrated that for complex anal fistula, the anal fistula plug strategy was associated with higher upfront costs than conservative medical management, but the cost of subsequent clinical gains was sufficiently low to confirm the cost-effectiveness of surgical intervention. In Chinese tertiary hospitals, the cost profile for T2DM patients is detailed as follows:

Single operation cost: approximately CNY: 10,000–20,000 (USD: 1,393–2,786/EUR: 1,209–2,418)
Hidden follow-up costs: The treatment cycle extends over several weeks to months in T2DM patients, requiring multiple follow-up visits for dressing changes and wound care, with cumulative follow-up costs of approximately CNY: 3,000–8,000 (USD: 418–1,114/EUR: 363–967)
Complication treatment costs: Additional medical expenses for postoperative infection, delayed healing, and anal incontinence, approximately CNY: 5,000–20,000 (USD: 696–2,786/EUR: 605–2,418)
Total long-term cost: No economic advantage in T2DM patients, due to extended healing time and high expenditure for complication management
Indications: Suitable for primary hospitals, patients with limited economic conditions, or simple low-lying fistulas with excellent preoperative glycemic control

(2) *Sphincter-Preserving Procedures (LIFT, TROPIS, EAF)*

Sphincter-preserving techniques demonstrate a favorable cost-effectiveness profile in T2DM patients despite higher upfront operation costs. The FIAT trial (33) demonstrated that the mean total cost was £2,738 (±£1,151) for the fistula plug group vs. £2,308 (±£1,228) for the surgeon's preference group, with Quality-Adjusted Life Years (QALYs) of 0.829 (±0.174) vs. 0.790 (±0.212), establishing a 35%–45% probability that the fistula plug is cost-effective at a willingness-to-pay threshold of £20,000–30,000 per QALY. In Chinese tertiary hospitals, the cost profile for T2DM patients is detailed as follows:

Single operation cost: approximately CNY: 15,000–30,000 (USD: 2,089–4,179/EUR: 1,814–3,627)

Core economic advantages in T2DM patients:

Postoperative healing time shortened by 30%–50% (2–4 weeks less than traditional procedures), significantly reducing follow-up and nursing costs
Markedly reduced complication rates (anal incontinence risk <5%), avoiding additional expenditure for adverse event treatment
Lower long-term recurrence risk, reducing the economic burden of reoperation and repeated treatment
Cost-effectiveness conclusion: The preferred cost-effective option for T2DM complicated with anal fistula
Indications: Suitable for most patients covered by medical insurance across various hospital settings, especially those with complex fistulas or high risk of poor wound healing

(3) *Novel Minimally Invasive/Biological Therapies (FiLaC, MSC Transplantation, CGF)*

Advanced biological therapies offer favorable clinical outcomes in high-risk T2DM patients, but require rigorous pre-treatment cost-benefit evaluation:

FiLaC laser treatment: approximately CNY 50,000–80,000 (USD 6,964–11,142/EUR 6,045–9,673), 3–5 times the cost of LIFT, with high costs driven by specialized laser equipment and disposable optical fibers
MSC transplantation: A single course of autologous MSC therapy exceeds CNY 10,000 (USD 1,393/EUR 1,209); allogeneic commercial products have significantly higher costs
Functional biomaterials: Tea tree oil-loaded slow-release hydrogel and CGF adjuvant therapy are not fully covered by medical insurance in most regions of China
Indications: Recommended only for T2DM patients at high risk of anal incontinence, with recurrent fistulas, or with failed previous traditional surgery; requires comprehensive evaluation based on economic status and treatment willingness

(4) *Metabolic Surgery (Roux-en-Y Gastric Bypass)*

For obese T2DM patients (Body Mass Index, BMI ≥30 kg/m²), metabolic surgery offers substantial long-term economic and clinical benefits. McGlone et al. (11) demonstrated that over 5 years, bariatric surgery was cost saving compared to best medical treatment (total cost £22,057 vs. £26,286, incremental difference £4,229) in patients with T2DM requiring insulin, driven by lower long-term hypoglycemic drug costs, reduced diabetes-related complication expenditure, and improved long-term health benefits. In Chinese tertiary hospitals, the cost profile is detailed as follows:

Initial surgical cost: approximately CNY 40,000–60,000 (USD 5,571–8,357/EUR 4,836–7,255)

Long-term economic benefits:

5-year diabetes remission rate reaches 42% (11)
Average annual savings on hypoglycemic drugs: CNY: 3,000–10,000 (USD: 418–1,393/EUR: 363–1,209)
Reduced fistula recurrence-related treatment costs: CNY: 5,000–15,000 (USD: 696–2,089/EUR: 605–1,814)
Cost break-even point: Approximately 3.5 years post-surgery (11)
Indications: Applicable only to T2DM patients with BMI ≥30 kg/m², poor glycemic control despite maximum medical therapy, and who meet the official surgical indications for metabolic surgery

#### 4.4.2 Clinical decision-making framework for T2DM patients

In clinical practice, a three-dimensional decision-making model of “efficacy–cost–patient tolerance” specific to T2DM patients should be established (10), with the following core principles:

Avoid excessive medical burden from over-pursuing minimally invasive/biological therapies without clear clinical indication, especially for T2DM patients with simple fistulas and good preoperative glycemic control
Be vigilant about elevated complication risks when selecting traditional surgical methods solely due to cost concerns, particularly in T2DM patients with impaired wound healing capacity and pre-existing diabetic peripheral neuropathy
Prioritize long-term cost-effectiveness rather than single-procedure costs alone, factoring in productivity losses, health-related quality of life, and long-term fistula recurrence risk in T2DM patients

Future multi-center cost-effectiveness analyses specific to T2DM complicated with anal fistula are warranted to provide evidence-based foundations for medical insurance policy formulation, clinical resource allocation, and individualized treatment selection for this high-risk population.

## 5 Current challenges and future research directions

### 5.1 Core clinical challenges

1. ***Lack of Diabetic-Specific Clinical Evidence***: Most existing clinical studies on anal fistula surgery exclude diabetic patients, and there is a lack of high-quality RCTs to verify the safety and efficacy of different surgical techniques, adjuvant therapies, and glycemic management regimens in this specific population.
2. ***Lack of Individualized Risk Stratification System***: There is currently no mature risk stratification system to predict the risk of postoperative infection, delayed healing, and recurrence in diabetic patients with anal fistula, which cannot guide individualized treatment decision-making.
3. ***Lack of Multidisciplinary Collaborative Management Standards***: There is no unified standard for multidisciplinary collaborative management of diabetic patients with anal fistula, and the communication and cooperation between anorectal surgery and endocrinology departments are insufficient in most primary medical institutions.

### 5.2 Future research directions

1. ***Diabetic-Specific Clinical Trials***: Large-sample, multicenter RCTs are needed to compare the long-term efficacy and safety of different sphincter-preserving surgical techniques in diabetic patients, and to verify the efficacy of regenerative medicine adjuvant therapies in this population.
2. ***Biomarker and Risk Stratification Research***: Studies are needed to identify biomarkers that can predict wound healing and fistula recurrence in diabetic patients, and to establish a validated risk stratification system based on glycemic control status, fistula anatomical complexity, and systemic inflammatory levels.
3. ***Novel Therapeutic Targets and Biomaterials***: Further research is needed on the molecular mechanism of fistula non-healing in diabetic patients, to develop targeted anti-inflammatory therapeutic agents and functional biomaterials that can adapt to the hyperglycemic wound microenvironment.
4. ***Standardized Multidisciplinary Management Guidelines***: National or international guidelines for the multidisciplinary management of diabetic patients with anal fistula should be developed, to standardize the perioperative glycemic management, surgical decision-making, and postoperative follow-up for this population.

## 6 Conclusion

T2DM is an independent risk factor for the onset and poor prognosis of anal fistula, and its core mechanism is the combined effect of metabolic disorder, immune dysfunction, microcirculation impairment, and gut microbiota dysbiosis. For diabetic patients with anal fistula, sphincter-preserving surgery is the core treatment strategy, and autologous MSC transplantation, functional biomaterials, and standardized perioperative glycemic management are important adjuvant therapies. Individualized multidisciplinary treatment strategies tailored to the patient's glycemic control status, fistula anatomical complexity, and anal function are essential to improve clinical prognosis. Future high-quality clinical studies specific to diabetic patients are warranted to optimize the treatment system for this population.
