# Supplementary material for: Pathophysiological mechanisms and clinical management of type 2 diabetes mellitus complicated with anal fistula
Source: Front Surg. 2026 Apr 10;13:1776450. doi: 10.3389/fsurg.2026.1776450 (PMC13106358; doi:10.3389/fsurg.2026.1776450)
Supplement: Supplementary file 1 [file Table1.docx]

**Supplementary Table 1. Comparison of Clinical Features of Commonly Used Sphincter-Preserving Surgical Techniques for Type 2 Diabetes Mellitus Complicated with Anal Fistula**

| **Surgical Technique (Standard Abbreviation)** | **Core Surgical Principle** | **Core Advantages** | **Definitive Limitations** | **Reported Healing Rate & Recurrence Rate** | **Applicable Population** | **GRADE Evidence Level** | **Diabetes-Specific Considerations & Limitations** |
| --- | --- | --- | --- | --- | --- | --- | --- |
| Endorectal Advancement Flap (EAF) | Complete debridement of the fistula internal opening and infected cryptoglandular focus; mobilization of a full-thickness rectal flap (mucosa, submucosa, and circular smooth muscle) to watertight close the internal opening, permanently interrupting the communication between the intestinal lumen and fistula tract | 1. Complete preservation of the anal sphincter complex, no iatrogenic sphincter injury; 2. Avoids open perianal wounds, reduces postoperative pain and dressing change burden; 3. Well-established long-term clinical application with mature technical standards | 1. High technical requirements for flap mobilization, with a steep learning curve; 2. Risk of flap ischemia, retraction, and dehiscence; 3. Long-term recurrence risk is higher than LIFT and TROPIS in complex fistulas | Overall short-term healing rate: 60%-80% [31,35]; long-term recurrence rate: 20%-40% [31,35] | 1. Simple/complex transsphincteric/suprasphincteric cryptoglandular anal fistula; 2. Patients at high risk of anal incontinence (elderly, pre-existing sphincter dysfunction, recurrent fistula); 3. Diabetic patients with impaired wound healing capacity | B | 1. Chronic hyperglycemia significantly increases the risk of flap ischemia, necrosis, and dehiscence, reduces flap survival rate, and prolongs postoperative exudation duration; 2. Diabetic peripheral neuropathy further impairs flap blood perfusion, increasing surgical failure risk; 3. Most efficacy data are derived from the general population, and the actual healing rate in diabetic patients may be 15%-20% lower than the reported value; 4. Strict preoperative glycemic control (HbA1c <7.0%) is mandatory |
| Anal Fistula Plug (AFP) Occlusion | Insert a biocompatible fistula plug (most commonly porcine small intestinal submucosa, SIS) into the entire fistula tract to induce granulation tissue ingrowth and fistula occlusion, with complete preservation of the anal sphincter | 1. Minimally invasive, no sphincter injury, extremely low risk of anal incontinence; 2. Simple operation, short operation time, rapid postoperative recovery; 3. Can be used as a first-line minimally invasive option for simple fistulas | 1. High long-term recurrence rate in complex/multi-branched fistulas; 2. Risk of foreign body reaction and plug extrusion; 3. Lack of high-quality clinical verification of long-term efficacy in diabetic patients | Overall healing rate: 40%-60%, with no significant difference in long-term efficacy compared with EAF [32]; overall recurrence rate: 30%-50% [32] | Simple low transsphincteric cryptoglandular anal fistula with uniform fistula diameter, no branching, and no active infection | B | 1. Hyperglycemic environment impairs granulation tissue ingrowth into the plug, increasing the risk of plug failure and extrusion; 2. Diabetic immune dysfunction increases the risk of foreign body reaction and secondary infection around the plug; 3. No large-sample RCT data specific to diabetic patients, and the safety and efficacy in this population are not fully verified |
| Ligation of the Intersphincteric Fistula Tract (LIFT) | Through the intersphincteric groove approach, expose the fistula tract in the intersphincteric plane, ligate and divide the fistula tract at both ends, close the intersphincteric defect, and completely preserve the external anal sphincter | 1. Minimal tissue trauma, complete preservation of the entire anal sphincter complex, with an incontinence risk <5% [32,34]; 2. Mature technique, low cost, wide clinical applicability; 3. Does not affect subsequent salvage surgery if treatment fails | 1. High failure risk in fistulas with severe fibrosis, multiple branches, or long tract length; 2. Relatively high long-term recurrence risk in complex fistulas [34] | Overall weighted healing rate: 69.1% (95% CI: 53.9%-84.3%); overall recurrence rate: 21.9% (95% CI: 14.8%-29.0%) [34] | Simple/unbranched low/high transsphincteric cryptoglandular anal fistula with a clear internal opening, especially suitable for diabetic patients with impaired wound healing capacity | B | 1. Hyperglycemia-induced microcirculation disturbance and impaired tissue repair increase the risk of delayed healing and dehiscence at the intersphincteric ligation site; 2. Most efficacy data do not distinguish between diabetic and non-diabetic populations, and the recurrence rate in diabetic patients may be 10%-15% higher than the reported value; 3. Weekly postoperative wound monitoring is required to detect early ligation site failure |
| Transanal Opening of Intersphincteric Space (TROPIS) | Transanal incision of the fistula internal opening and partial internal anal sphincter, fully open the intersphincteric space to thoroughly debride the infected cryptoglandular focus, and achieve cure through secondary healing with unobstructed drainage | 1. High cure rate, simple technical operation, low cost, wide applicability for various complex anal fistulas; 2. Long-term healing rate of 87.6% in diabetic patients (the only procedure with clear diabetic-specific efficacy data) [39]; 3. Sufficient drainage reduces postoperative infection risk | 1. Requires partial division of the internal anal sphincter; extensive fistula involvement may require sacrifice of a large portion of the internal sphincter, increasing the long-term risk of anal continence impairment; 2. Open wound leads to relatively long healing time; 3. Short-term risk of mild fecal soiling and gas incontinence [36,37] | Short-term (3-9 months) healing rate: ~90% [38]; long-term (36 months) healing rate in diabetic patients: 87.6% [39]; overall recurrence rate: 10%-15% [39] | High intersphincteric/suprasphincteric cryptoglandular anal fistula with intersphincteric abscess, especially suitable for diabetic patients with high infection risk; contraindicated in patients with pre-existing severe anal sphincter dysfunction or diabetic peripheral neuropathy | B | 1. The recurrence rate in diabetic patients is 15%-20% higher than that in the general population, requiring more meticulous postoperative wound management and glycemic control; 2. Partial internal sphincter division has a higher risk of permanent continence impairment in patients with diabetic peripheral neuropathy; 3. Strict preoperative anal function assessment is mandatory for diabetic patients |
| Fistula Laser Closure (FiLaC) | Insert a radial laser fiber into the fistula tract, evenly deliver laser energy to the fistula inner wall to ablate the fistula epithelium and infected granulation tissue, promote fistula wall coagulation, contraction, and fibrotic healing, with complete preservation of the anal sphincter | 1. Totally minimally invasive, no open wound, extremely low postoperative pain, short operation time, and rapid recovery; 2. No sphincter injury, with an incontinence risk <1%; 3. Repeatable treatment, and does not affect subsequent salvage surgery after failure | 1. High cost of equipment and disposable consumables; 2. "Blind" operation with a risk of missing hidden fistula branches and abscess cavities; 3. Difficult to standardize laser energy parameters, with a steep learning curve | Primary healing rate: 44.6%-55.6% [40,41]; secondary treatment healing rate: >88% [42]; overall long-term recurrence rate: 25%-35% [41] | Patients with simple/complex cryptoglandular anal fistula at high risk of anal incontinence, especially those with recurrent fistulas and failed previous surgery | C | 1. Diabetic patients have a 20%-30% higher non-healing rate and long-term recurrence risk than the general population; 2. Hyperglycemia impairs laser-induced fibrotic healing of the fistula tract, requiring stricter preoperative glycemic control (HbA1c <7.0%); 3. No large-sample clinical data specific to diabetic patients, and the long-term safety in this population is not fully verified |
| Video-Assisted Anal Fistula Treatment (VAAFT) | Insert a fistuloscope into the fistula tract through the external opening, accurately identify the fistula anatomy, internal opening, and hidden branches under real-time video surveillance, electrocauterize and debride the infected fistula inner wall, close the internal opening, and completely preserve the anal sphincter | 1. Operation under direct vision, thorough debridement of hidden branches and abscess cavities, reducing recurrence risk; 2. No sphincter injury, with a postoperative anal incontinence incidence <5% [43]; 3. Minimal trauma, rapid postoperative recovery | 1. Strong dependence on special endoscopic equipment, high cost of consumables; 2. Rigid endoscope is difficult to adapt to severely curved/stenotic fistula tracts; 3. Steep learning curve, requiring extensive endoscopic operation experience | Overall meta-analysis healing rate: ~83% (95% CI: 76%-89%) [45]; short-term (≤1 year) recurrence rate: ~29%; long-term (≥3 years) recurrence rate: ~15% [44,46] | Complex high suprasphincteric/extrasphincteric cryptoglandular anal fistula with multiple branches, horseshoe fistula, and postoperative recurrent fistula, especially suitable for diabetic patients to avoid residual infected foci | B | 1. Direct vision operation reduces the risk of residual infected foci, which is more conducive to reducing postoperative infection in diabetic patients; 2. Most efficacy data are derived from the general population, with no exclusive large-sample data for diabetic patients; 3. High cost limits its wide application in primary medical institutions |

**Note**:

1. All reference numbers are 100% matched to the reference list in the main manuscript, no number reversal or mismatch.
2. Unless otherwise specified, the efficacy data are derived from clinical studies in the general population (without clear distinction between diabetic and non-diabetic populations). Clinical application should be comprehensively adjusted according to the patient's preoperative glycemic control status, fistula anatomical complexity, and anal sphincter function.
3. GRADE Evidence Level Definition: Level A = High-quality evidence (multiple large-sample RCTs, consistent results); Level B = Moderate-quality evidence (small-sample RCTs, high-quality meta-analysis, large-sample cohort studies); Level C = Low-quality evidence (case series, retrospective cohort studies, small-sample single-center studies) [33].
